# Prevalence of childhood eczema and food sensitization in the First Nations reserve of Natuashish, Labrador, Canada

**DOI:** 10.1186/1471-2431-14-76

**Published:** 2014-03-20

**Authors:** Robert GP Forsey

**Affiliations:** 1 Discipline of Family Medicine, Memorial University of Newfoundland, Labrador-Grenfell Health, Happy Valley-Goose Bay, Newfoundland and Labrador, Canada

## Abstract

**Background:**

The Mushua Innu of Natuashish, Labrador, Canada seem to have a high rate of childhood eczema. Anecdotally this problem seems to be more common now than 20 years ago. There has been speculation that this could be related to food sensitization that may have arisen coincident with a move away from a traditional Innu diet. We undertook to assess the prevalence and severity of pediatric eczema in Natuashish (population 792), and investigate the level of sensitization to common food antigens.

**Methods:**

Over a three-month period we performed a population survey of all children in the community from the ages of 2–12 inclusive. The one-year prevalence of eczema was assessed using the United Kingdom Working Party’s diagnostic criteria, and graded on the Nottingham Severity Scale. All children with eczema and twice as many age/sex matched controls were offered complete blood counts, total IgE, and food specific IgE levels for egg white, cow’s milk protein and wheat.

**Results:**

One hundred and eighty two (95% of the eligible children) were assessed. Of the 182 children examined eczema was diagnosed in 30 (16.5%) - 22 females and 8 males. The majority of children with eczema (20/30) were classified as being in the moderate and severe category. Of the 22 with eczema and 40 controls who consented to venipuncture all but 3 had IgE levels above the lab's reference range. Food specific antibody assays showed that 32, 23, and 5 percent of children with eczema were sensitized to egg, milk, and wheat respectively. None of the controls were sensitized.

**Conclusions:**

The children of Natuashish, Labrador have a high rate of eczema, much of it graded as moderate or severe. IgE levels were markedly elevated in children with and without eczema, with average values at least ten-fold higher than other populations. There is no evidence of an unusual amount of sensitization to egg, milk or wheat.

## Background

Eczema is a chronic relapsing disease that is characterized by erythematous pruritic skin lesions. Many factors affect its prevalence and severity. Susceptibility genes express a defective barrier protein (filaggrin), which increases epidermal permeability and water loss [1,2]. The immune response to this permeability is affected by infectious and environmental factors [2,3]. Its economic burden is considerable, and caregiver stress can exceed that of caring for a child with diabetes [4]. Population surveys using the ISAAC protocol [5] show rates that vary widely- as low as 2% in China and eastern European countries, 8.5% in Canada, and 15.9% in Japan. The picture is fluid; some high prevalence countries have shown a decrease, while many developing countries with a low prevalence have experienced increases [6,7]. Prevalence in the former East Germany rose to equal that of West Germany after reunification [8]- too quickly to be explained by gene frequency changes, but coinciding with a more “Western” diet and other social changes.

Much time has been spent examining potential influences- the literature is complex and at times contradictory. Dietary and environmental changes parallel the rising rate of eczema although causality remains unproven [9]. Gender, nutrition, number of siblings, allergic status, exposure to acetaminophen or antibiotics, vitamin D and climate have been examined [10-13]. Indoor exposure to dust, animal dander, molds, tobacco smoke, heating systems and aeroallergens may also play a role [14,15]. Evidence is contradictory about the role of breastfeeding [16,17]. Western and urban populations tend to have more eczema than those that are oriental and rural [5]. Less exposure to childhood infections may cause higher rates of atopic disease (the Hygiene Hypothesis) [18].

The connection between atopy and eczema has been debated- the link is stronger in severe (hospitalized) patients and weaker in the community setting [19], stronger in affluent countries and weaker in developing ones [20]. One early study noted elevated IgE levels in 43% of patients with eczema [21], and another in 2004 noted higher IgE levels in severe cases [22]. Eczematous children commonly have food sensitization- 40% with moderate/severe eczema have food allergies [23]. Milk and eggs can provoke flares in infants and some adolescents [24]. The 2007 GA^2^LEN/EAACI recommendation [25] to consider food triggers is noteworthy, and there are plausible mechanisms to implicate IgE in chronic inflammation [26]- in addition to its well-known role in acute hypersensitivity.

Little Canadian research has been done, although a 1999 questionnaire compared prevalence rates in the Canadian cities of Saskatoon, Saskatchewan (17.3%) and Hamilton, Ontario (15.4%) [27]. It is worth noting that the children in this study were not examined, and that reported rates in surveys can be much higher (even double) rates observed in studies that include a clinical assessment [28].

There is limited information on eczema in circumpolar and First Nations communities. Sami children had a higher rate than their Norwegian schoolmates [29], and affluent Norwegian children had more than Russians [30]. Inuit schoolchildren in northern Quebec had low rates of exercise induced asthma and atopy, although eczema rates were not assessed in this study [31].

This project was designed to assess the prevalence of childhood eczema in Natuashish, and the level of sensitization to foods now common in the diet of the Innu.

## Methods

### Data source and population

The Mushua Innu of Labrador led a nomadic existence until 1967 when they were settled into the community of Davis Inlet. The entire community relocated to the newly constructed town of Natuashish in 2004 in hopes of improving housing and basic community services. Health care to the 725 inhabitants of Natuashish is provided by a station staffed by three nurses, with regular visits from a physician based in Goose Bay.

Community members and clinicians noted a lot of eczema, apparently more than in other coastal communities. Nurses reported seeing fewer skin infections but more eczema over the past decades. The emergence of this problem over two decades does parallel the adoption of a less traditional, more “Westernized” diet. Traditional foods for the Innu would include caribou meat, fish and berries, with little milk, egg and less flour.

The Mushua Innu Health Commission and the band council requested further investigation. Memorial University of Newfoundland’s Human Investigations Committee, the Labrador-Grenfell Health Research Review Committee, and the Mushua Innu Band Council approved the project. It was conducted in accordance with the Canadian Institutes of Health Research: Guidelines for Health Research involving Aboriginal People and the Tri-Council Policy Statement: Ethical Conduct for Research Involving Humans. Consent discussions, questionnaires and interviews were offered in either Innuamun or English, and written informed consent was obtained from the children’s parents/guardians.

### Assessment of prevalence and severity of eczema

The study was publicized through the clinic and local radio station. Community birth and public health records were used, and all children were identified. Over a three-month period (June-August 2008) those between the ages of 2 and 12 years were assessed. The one-year prevalence of eczema was established using the United Kingdom Working Party’s (UKWP) diagnostic criteria [32] (see section ‘‘United Kingdom Working Party’s diagnostic criteria’’). We assessed the one-year (rather than the point) prevalence because eczema is typically an evanescent condition-examination alone will miss some who are currently in remission. This approach improves sensitivity [33,34]. Eczema was graded using the Nottingham Severity Score [35].

### Assessment of eosinophil, total and food specific IgE levels

All thirty children with eczema were offered a complete blood count (CBC), total and food specific IgE (FSIgE) levels for egg white, cow’s milk protein, and wheat. Twenty-two consented, and 40 age and sex matched controls were likewise tested.

Blood samples were collected in Natuashish. CBC’s were run at the Labrador Health Center in Goose Bay. Serum was frozen and transported to the immunology laboratory at the Health Sciences Center, St. John’s for total IgE and FSIgE levels (IMMULITE® 2500 -Siemens).

As per standard we accepted FSIgE levels of ≥0.35 kIU/L to indicate sensitization. This cutoff is far below that required to make a diagnosis of food allergy (6 kIU/L-eggs, 32 kIU/L-milk, and > 100 kIU/L-wheat) [36,37].

### United Kingdom Working Party’s diagnostic criteria

To meet the UKWP diagnostic criteria, the child must have: a history of an itchy skin condition in the last 12 months plus three or more of the following:

•a history of a rash in the skin creases (folds of elbows, behind the knees, front of ankles or around the neck);

•a personal history of asthma or hay fever (or history of atopic disease in a first degree relative for children under 4 years of age);

•a history of generally dry skin in the last year;

•onset under the age of two years;

•visible flexural dermatitis (or dermatitis of cheeks and the outer aspects of limbs in children <4 years) as determined on clinical exam.

### Statistical analysis/comparison with other populations

We used SPSS Statistics v17.0. Rates from four other prevalence studies were compared with our rate using two tailed paired Fisher’s Exact Test. Laboratory results were compared using Student’s t-test.

## Results

Virtually all children who were living in Natuashish that summer were assessed. Census data suggests there were approximately 192 children in that age range, and we saw 182. Examination findings are summarized in Table 1. Thirty children met the UKWP diagnostic criteria for eczema. The one-year prevalence rate was 16.5%. The proportion of females with eczema (22 out of 102) was significantly higher than the proportion of males (8 out of 80). Forty-three percent (13 children) of the eczema cases were severe, 23% (7) were moderate, and the remaining cases were mild. Table 2 summarizes the laboratory results-all but 3 children (in both eczema and control groups) had elevated total IgE levels. Food specific antibody assays showed that 32, 23, and 5 percent of children with eczema were sensitized to egg, milk, and wheat respectively. None of the controls were sensitized.

**Table 1:** Clinical assessment of 182 children in Natuashish

**Age in years**	
Mean (SD)	6.35 (2.9)
Female sex	
# females (%)	102 (56)
Eczema present (UKWP criteria)	
# (%)	30 (16.5)
Females with eczema*	
# eczema/total # of females (%)	22/102 (21.6)
Males with eczema*	
# eczema/total # of males (%)	8/80 (10)

*females were significantly more likely to have eczema than males (p = 0.037).

**Table 2:** Laboratory comparison of eczema and control groups

		**Eczema** ** (N = 22)**	**Eczema-free controls** ** (N = 40)**	**p value (where applicable)**
Age in years: Mean (SD)		5.48 (2.50)	5.37 (2.29)	
Female sex: # (%)		14 (64)	31(77.5)	
Eosinophil count: (SD) (×10^9^/L)				
		0.73 (0.08)	0.96 (0.14)	0.245
IgE levels*: (kIU/L) # (%)	0-52	0 (0)	2 (5)	
	53-199	3 (13.6) ^§^	10 (25) ^§^	
	200-1999	10 (45.5)	22 (55.0)	
	≥2000	9 (40.9)	6 (15.0)	
Food specific IgE: (kIU/L) Mean (SD)	Egg	2.7 (6.4)	0.2 (0.5)	0.010
	Milk	0.29 (0.61)	0.00 (0.02)	0.005
	Wheat	0.57 (2.53)	0.00 (0.02)	0.160
# of children with food-specific antibody levels ≥ 0.35 kIU/L: # (%)	Egg	7 (32)	0 (0)	
	Milk	5 (23)	0 (0)	
	Wheat	1 (5)	0 (0)	

*Normal IgE: Age 2–9 years = 0–52 kIU/L, Age >9 years = 0–199 kIU/L.

^§^All but one of these 13 children were in the 2–9 year age group, thus 12/13 had IgE levels above the age appropriate reference range.

## Discussion

Our 16.5% prevalence is higher than reported in Australia [38], Italy [39], London [34] and Scotland [40] and similar to high prevalence areas like Japan [41] (Table 3). Our female/male ratio of 2:1 was higher than the 1.2:1 ratio reported elsewhere [28,42]. Sex related differences in eczema subtypes have been reported-eczema in boys has been more often associated with sensitization to various allergens (extrinsic) whereas girls were more likely to be intrinsic [43].

**Table 3:** Eczema rates (UKWP diagnostic criteria)-a comparison between Natuashish and other pediatric populations

**Location**	**One year eczema prevalence**	**p value**
Natuashish	16.5	
Age 2-12		
Australia (1999) [38]		
N = 2491	10.8	0.027*
Age 4-18		
Italy (2003) [39]		
N = 1369	6.9	0.0001*
Age 9		
London (1996) [34]		
N = 877	8.5	0.002*
Age 3-11		
Japan (2007) [41]		
N = 3849	15.4	0.600
Elementary school students (first and sixth graders)		
Lothian, Scotland (1996) [40]		
Number unspecified	8.1	N/A
Age 2-11		

*The Australian, Italian and London studies all had significantly lower rates than Natuashish.

A high number (67%) of the children with eczema were in the moderate or severe category. The Danish DARC study [44] of children with eczema showed 43% were in the moderate-severe categories, and a Japanese study [45] graded 13-19% of their cases as moderate-severe. Two Australian studies reported rates of 36 and 13 percent [38,46].

An unexpected finding was elevated levels of IgE in all but three of the 62 subjects (those with eczema and those in the eczema-free control group). This finding was verified with repeat samples. The lab performed no dilutions beyond 2000 kIU/L so it is impossible to calculate mean IgE levels for both groups. That being said-the lowest these means could possibly have been were 1247 kIU/L in the eczema group and 685 kIU/L in the controls. These two figures were arrived at by assuming a best-case scenario in which the 15 samples reported as “>2000 kIU/L” were 2001 kIU/L (one unit above the cut-off). These two numbers contrast markedly with typical population means and medians that are in the range of 18–122 kIU/L [47-50]. Even atopic children are usually in the 250–380 range [47,49]. There is no obvious cause for this-parasitic infections are not common in Natuashish, and normal eosinophil counts suggest this explanation is unlikely. One study of Inuit children in Alaska found that 17% had IgE levels > 1000 kIU/L, although their mean was still only 122 kIU/L [51]. There is also a report of unexplained hyperimmunoglobulinemia E (mean 11,850 kIU/L) in the Haurorania Indians of Ecuador [52]. Neither the Alaskan children nor the Ecuadorians showed high rates of conditions that are associated with elevated IgE levels such as atopic disease, dermatitis, B cell neoplasia, hypersensitivity reactions and parasites.

In our study children with eczema showed sensitization rates comparable to those reported elsewhere. On average 50% of children and 35% of adults with eczema are sensitized to common allergens [53]. Sensitization is in fact common in the general population-unselected children in Denmark [44,54], Greenland [49], Russia and Finland [55] showed rates ranging from 0-14%.

### Limitations

There would be children who were missed in this study. Community members do travel back and forth to visit other communities in Labrador and northern Quebec. Eczema severity scoring may have been affected by the fact that the children’s caregivers (not the examiner) indicated the extent of skin involvement on the diagram. Nevertheless, the 30 children with eczema had it for an average of six months per year and averaged three nights per week of interrupted sleep so many of these cases were not mild. We also limited the number of antigens tested-others that may contribute to eczema were not formally studied (although subsequent testing did show that approximately 25% of the children with eczema were sensitized to house dust mite and cat dander). We were unable to explore the possibility of non-IgE mediated food triggers, although food antigen specific T cells and other inflammatory mediators can play a role [1,56].

The fact that food sensitization coexists with eczema does not necessarily prove a cause and effect relationship-monosymptomatic, late phase eczematous reactions after food ingestions are not common [44], and food sensitization cannot always be linked to a worsening of the eczema [54]. Inappropriate use of elimination diets may cause other health problems. A recent Cochrane meta-analysis of the use of exclusion diets to improve established eczema [57] found little evidence to support the practice, although it is notable that subjects in eight out of the nine studies in this analysis were not assessed for possible food sensitization-the one study of infants with known egg sensitization [58] did show an improvement when an elimination diet was instituted.

## Conclusions

The issue of eczema in Natuashish is a substantial public health concern, with a high one-year prevalence and many moderate-severe cases. Many more females than males were affected. Unexpectedly, IgE levels exceeded the normal range in 59 of the 62 eczematous and non-eczematous children, many by a factor of tenfold. Sensitization rates in the group of children with eczema were comparable to what has been observed in other studies.

## Competing interests

The author declares that he has no competing interests.

## Pre-publication history

The pre-publication history for this paper can be accessed here:

http://www.biomedcentral.com/1471-2431/14/76/prepub
